# Comparative Analyses by Sequencing of Transcriptomes during Skeletal Muscle Development between Pig Breeds Differing in Muscle Growth Rate and Fatness

**DOI:** 10.1371/journal.pone.0019774

**Published:** 2011-05-26

**Authors:** Xiao Zhao, Delin Mo, Anning Li, Wen Gong, Shuqi Xiao, Yue Zhang, Limei Qin, Yuna Niu, Yunxue Guo, Xiaohong Liu, Peiqing Cong, Zuyong He, Chong Wang, Jiaqi Li, Yaosheng Chen

**Affiliations:** 1 State Key Laboratory of Biocontrol, Sun Yat-sen University, Guangzhou, Guangdong, People's Republic of China; 2 College of Animal Science, South China of Agricultural University, Guangzhou, Guangdong, People's Republic of China; Laboratoire Arago, France

## Abstract

Understanding the dynamics of muscle transcriptome during development and between breeds differing in muscle growth is necessary to uncover the complex mechanism underlying muscle development. Herein, we present the first transcriptome-wide longissimus dorsi muscle development research concerning Lantang (LT, obese) and Landrace (LR, lean) pig breeds during 10 time-points from 35 days-post-coitus (dpc) to 180 days-post-natum (dpn) using Solexa/Illumina's Genome Analyzer. The data demonstrated that myogenesis was almost completed before 77 dpc, but the muscle phenotypes were still changed from 77 dpc to 28 dpn. Comparative analysis of the two breeds suggested that myogenesis started earlier but progressed more slowly in LT than in LR, the stages ranging from 49 dpc to 77 dpc are critical for formation of different muscle phenotypes. 595 differentially expressed myogenesis genes were identified, and their roles in myogenesis were discussed. Furthermore, *GSK3B*, *IKBKB*, *ACVR1*, *ITGA* and *STMN1* might contribute to later myogenesis and more muscle fibers in LR than LT. Some myogenesis inhibitors (*ID1*, *ID2*, *CABIN1*, *MSTN*, *SMAD4*, *CTNNA1*, *NOTCH2*, *GPC3* and *HMOX1*) were higher expressed in LT than in LR, which might contribute to more slow muscle differentiation in LT than in LR. We also identified several genes which might contribute to intramuscular adipose differentiation. Most important, we further proposed a novel model in which *MyoD* and *MEF2A* controls the balance between intramuscular adipogenesis and myogenesis by regulating *CEBP* family; *Myf5* and *MEF2C* are essential during the whole myogenesis process while *MEF2D* affects muscle growth and maturation. The *MRFs* and *MEF2* families are also critical for the phenotypic differences between the two pig breeds. Overall, this study contributes to elucidating the mechanism underlying muscle development, which could provide valuable information for pig meat quality improvement.

The raw data have been submitted to Gene Expression Omnibus (GEO) under series GSE25406.

## Introduction

Lean and obese pig breeds have significant genetic difference in muscle growth rate and fatness. Landrace (LR), an improved lean pig breed, is characterized by high lean meat percentage, fast-growing muscle and high body weight [1], [2], [3]. In contrast, Lantang (LT) is a China indigenous obese pig breed, characterized by high intramuscular fat content, slow-growing muscle and low body weight [1], [4]. Differences in the terms of muscle growth between LR and LT are thus a potentially good model for studying the mechanism underlying muscle development and phenotypes differences. The research of complex mechanism underlying muscle development is beneficial to genetic improvement for lean meat percentage and meat quality. Moreover, understanding the complex mechanism underlying muscle development could contribute to understanding human muscle regeneration and muscular atrophy, since the pigs are similar to humans in physiological, pathological and genomic characteristics [3], [5].

Muscle development is a complex process. The myoblasts are the myogenic progenitor cells which originate from mesenchymal precursor cells and develop into multinucleated muscle fibers [3], [6]. It is temporally ordered into four steps: (1) the determination of myogenic progenitor cells (myoblasts), (2) the proliferation of myoblasts (3) differentiation and fusion of myoblasts into multinucleated myotubes and myofibers, and (4) growth and maturation of muscle until postnatal [7], [8], [9], [10]. In pig, the postnatal muscle growth is largely determined by the total number of fibers (TNF), which is determined by two major waves of fiber generation before birth. The first wave happens at 35–60 dpc (days post coitus), and the second at 54–90 dpc [9], [11]. Therefore, the muscle growth is predominantly determined during prenatal skeletal muscle development [3], [9], [12]. However, some reports suggested that there existed the third muscle development wave [9], [13] and a transition of slow-oxidative to fast-glycolytic fiber types from birth until 60 dpn (days post natal) [14].

Previous studies have identified several genes that positively or negatively regulate myogenesis using single major gene studies. The most important of these genes are the myogenic regulatory factors (*MRFs*) involving myogenic differentiation 1 (*MyoD*), myogenic factor 5 (*Myf5*), myogenin (*MYOG*) and *MRF4* (*Myf6*). *Myf5* and *MyoD* are myogenic determination factors contributing to myoblast specification and differentiation, while *MYOG* and *MRF4* are myogenic differentiation factors contributing to the induction of terminal differentiation [15], [16], [17]. In addition, *Myf5* regulates myoblasts proliferation and *MYOG* is related to birth weight and growth rate in mammals [18]. However, some studies have revealed that MRFs are not altered during porcine muscle development, and myogenesis in the pig might depend on the balance of differentiation-stimulating and differentiation-inhibiting factors [6], [19]. In order to comprehensively understand the mechanism underlying porcine muscle development, expression profile analyses of prenatal skeletal muscle have been performed using Microarray or SAGE [3], [6], [19], [20], [21]. However, drawbacks of microarray include background interference/cross-hybridization and the ability only to measure the relative abundance of predefined transcripts. SAGE analysis is limited by laborious and costly cloning and sequencing steps [22]. So only a relatively small number of myogenesis genes could be analyzed [3], [6], [19], [20], [21], [23], [24]; the resolution of both technologies was too poor to analyze the low expression genes. Recently, the Solexa/Illumina Genome Analyzer, a second generation sequencing technology, has facilitated complex transcriptome studies because Digital gene expression (DGE) can analyze transcriptomes without either predefined transcripts or laborious cloning steps. DGE is a high throughput and ultra-deep sequencing technology with major advances in terms of robustness, resolution, comparability and richness, and has been used for transcriptome analysis in recent years [22].

Previous reports have made inroads in terms of understanding the mechanism underlying muscle development, but several issues including the regulatory network of muscle development require further exact and comprehensive study. In this study, DGE was used to investigate the skeletal muscle transcriptomes of LT and LR at 35, 49, 63, 77, 91 dpc and 2, 28, 90, 120, 180 dpn. In addition, morphological differences between muscle samples were studied using histological sections. This research aimed to comprehensively analyse the mechanisms underlying muscle development, and reveal the muscle development differences between the two breeds from embryo to adult.

## Results

### Histological appearance and fiber sizes

**Figure 1:**
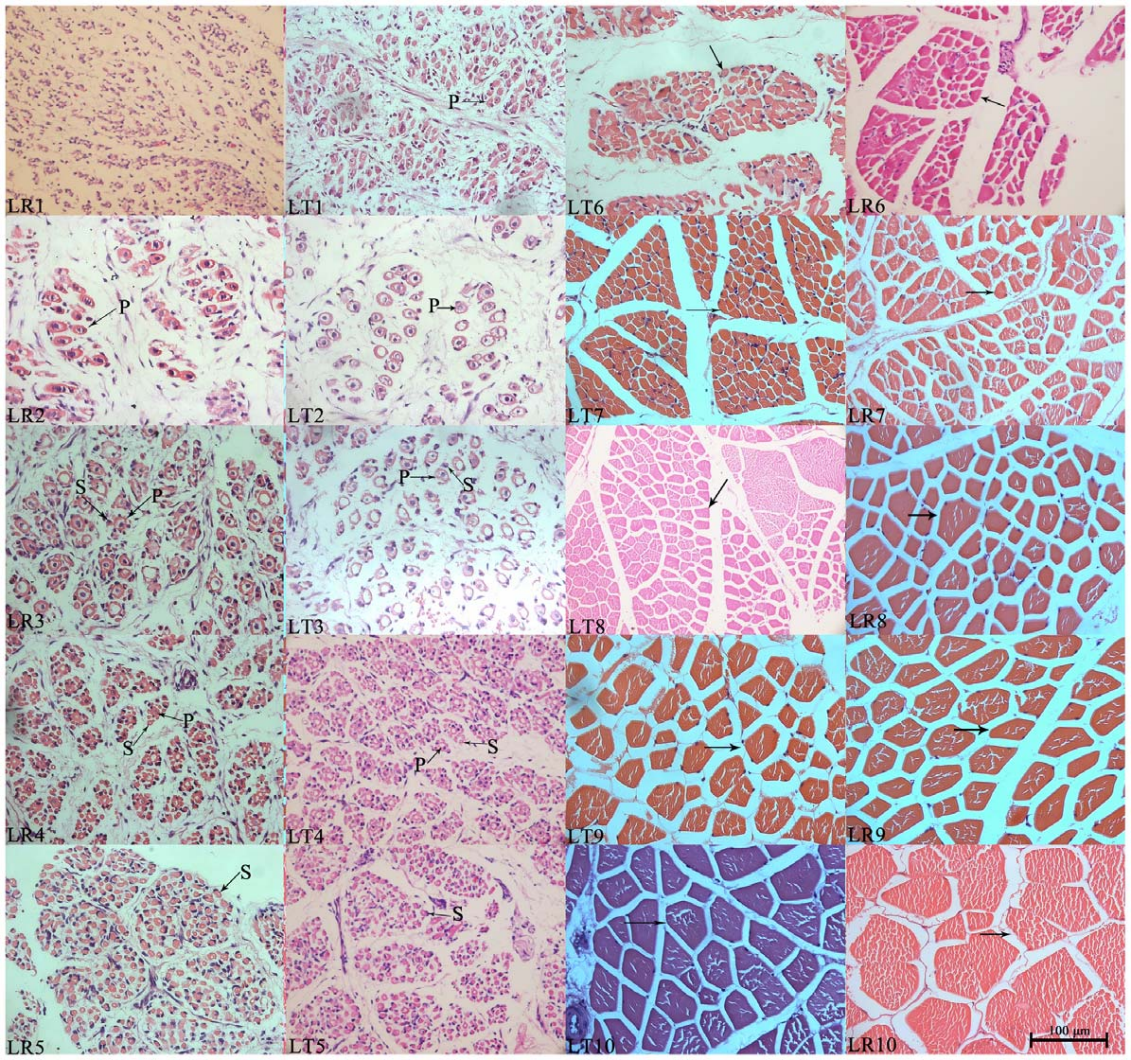
Morphological variations of longissimus muscle samples during development and between breeds. LR indicates Landrace and LT indicates Lantang. Numbers (1–10) indicate distinct developmental stages including 35 (1), 49 (2), 63 (3), 77 (4), 91 (5) days post coitus (dpc) and 2 (6), 28 (7), 90 (8), 120 (9), 180 (10) days post natum (dpn). Arrows point to myofibers or muscle fibers, P indicates primary fiber and S indicates secondary fiber. All areas were photographed at a magnification of ×400.

Morphological differences among muscle samples were studied by histological section (Figure 1). At 35 dpc, few primary fibers were present in LT but not in LR. The cross-section of presumptive primary fibers observed at 35 dpc enlarged at 49 dpc, and the fiber bundles were isolated by connective tissue in both breeds. The secondary fibers formed around primary fibers at 63 dpc and gradually increased until 91 dpc; more fibers were found in LR. At 91 dpc, it was difficult to distinguish primary fibers from secondary fibers and presumptive muscle bundles began to emerge. Moreover, muscle fiber diameter of LR is larger than LT during this period (Figure 1, LT5 and LR5). During postnatal development, the muscle phenotypes still changed from 91 dpc to 28 dpn. The muscle fibers grew more rapidly and larger cross-section areas were found in LR than in LT (Figure 1, LT6–LT10 and LR6–LR10).

### Solexa sequencing and gene annotation

Based on the sequencing 20 libraries of muscle samples, 4.22±0.9×10^6^ tags with 5.0±0.18×10^5^ distinct tags were obtained from each sample on average. After filtering adaptor tags, empty reads, low quality tags and one-copy tags from these tags, 3.84±0.9×10^6^ total clean tags (quantity of all the clean tags sequenced) with 1.5±0.5×10^5^ distinct clean tags (the type quantity of different clean tags) were remained (Table S1). Saturation analysis demonstrated that newly emerging distinct tags were gradually reduced as the total number of sequence tags rose, and the library capacity approached saturation when the number of sequencing tags reached 2.5 million (Figure S2). In addition, the heterogeneity and redundancy of mRNA were confirmed, which demonstrated that high copy number clean tags (copy number more than 100) accounted for 65.2% of the total clean tags but less than 3% of the clean distinct tags. In contrast, low copy number clean tags (copy number less than 5) accounted for approximately 4.3% of the total clean tags but more than 60% of the clean distinct tags (Figure S1).

For tag mapping, approximately 61,614 sequences were obtained from *Sus Scrofa* RefSeq and UniGene (NCBI36.1, 20090827) databases, of which 55,490 had the CATG site and all CATG+17 tags were used as reference sequences. In the present study, 189,160 total reference sequences with 149,321 unambiguous sequences (the sequences matched only to one gene) were obtained. Approximately 78.9% of the total clean tags and 53.26% of the distinct clean tags were mapped to the reference tag database, while 35.3% of total clean tags and 34.77% of distinct clean tags were mapped unambiguously (tags mapped to only one gene) to the reference tag database (Figure S3). CATG position analysis indicated that most tags matched to the 1st or 2nd 3′ CATG site in high-confidence transcripts (Figure S2). Saturation analysis demonstrated that the number of newly mapped genes fell gradually as the number of total sequence tags increased and approached saturation when it reached 1 million (Figure S2). In addition, gene expression analysis suggested that most genes were expressed at very low levels (Figure S2). In this study, 40% and 13.2% clean distinct tags mapped to sense genes and antisense genes, respectively.

### Cluster analysis and correlation analysis of 20 skeletal muscle libraries

**Figure 2:**
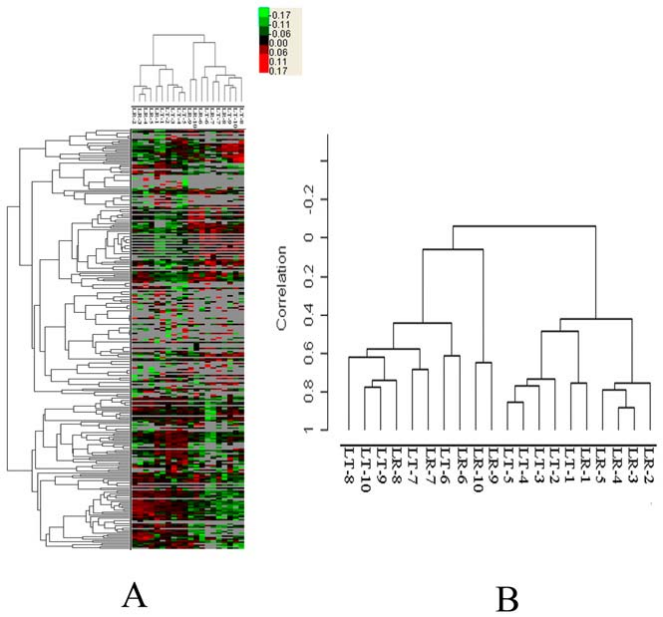
Similarity of transcriptome profiles between 20 skeletal muscle libraries. (A) Correlation analysis of 20 libraries by Pearson's correlation coefficient. (B) Systematic cluster analysis of 20 libraries by Cluster 3.0 and TreeView software.

Systematic cluster analysis (using myogenesis genes) and correlation analysis (using all genes) were performed to compare the relationship among 20 skeletal muscle libraries. Both analyses produced similar results, indicating that the 20 different transcription profiles could be divided into several distinct classes (Figure 2).

### Transcriptome analysis between different developmental stages

#### Temporal analysis of differentially expressed genes

The number of temporally differentially expressed (DE) genes with log_2_ ratio>0.5 (*P*<0.009, *FDR*<0.02) between libraries are presented in Table 1. Totals of 9624 and 8554 DE genes were identified during muscle development in LR and LT, respectively (Table 1, Table S2). There were more DE genes during the early stages of prenatal (35 and 49 dpc) and postnatal (from 91 dpc to 28 dpn) development than other stages (Table 1, Table S2).

**Table 1:** The numbers of differentially expressed genes between libraries.

The comparison between different libraries	numbers of differentially expressed genes					
	Total DE genes	Total DE genes-up	Total DE genes-down	MD related DE genes	MD related DE genes-up	MD related DE genes-down
LR-49 dpc/35 dpc	2461	876	1585	220	109	111
LR-63 dpc/49 dpc	462	179	282	87	58	29
LR-77 dpc/63 dpc	241	110	131	66	36	30
LR-91 dpc/77 dpc	423	256	167	63	35	28
LR-2 dpn/91 dpc	2006	771	1235	220	112	108
LR-28 dpn/2 dpn	1269	285	984	149	94	55
LR-90 dpn/28 dpn	678	235	443	129	51	78
LR-120 dpn/90 dpn	1373	808	565	178	56	122
LR-180 dpn/120 dpn	702	247	455	85	44	41
LT-49 dpc/35 dpc	1362	863	499	147	100	47
LT-63 dpc/49 dpc	702	341	361	108	68	40
LT-77 dpc/63 dpc	330	185	145	68	35	33
LT-91 dpc/77 dpc	316	163	153	68	44	24
LT-2 dpn/91 dpc	2301	1067	694	271	93	178
LT-28 dpn/2 dpn	1453	1036	417	199	71	128
LT-90 dpn/28 dpn	1036	573	463	134	66	68
LT-120 dpn/90 dpn	642	357	285	97	57	40
LT-180 dpn/120 dpn	403	124	279	90	51	39

LR, Landrace; LT, Lantang; MD, muscle development; DE, differentially expressed; up, up regulation; down, down regulation.

#### Gene Ontology (GO) analysis of temporal DE genes

**Figure 3:**
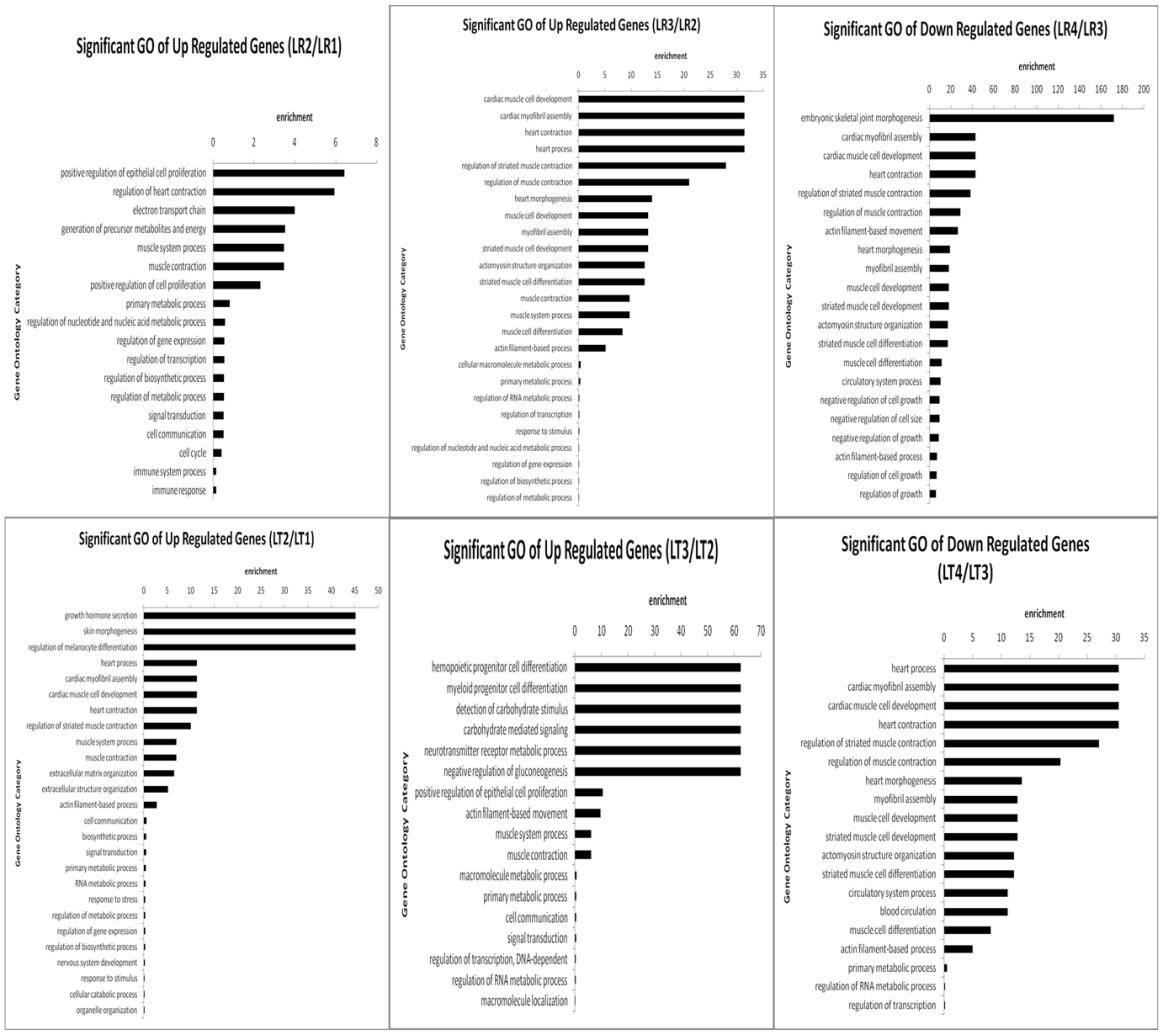
The muscle-related biological processes of differentially expressed genes during different muscle development stages. (**A**) Muscle development-related GO categories were up-regulated at 49 and 60 dpc and down-regulated at 77 dpc for Landrace. (**B**) Muscle development-related GO categories were up-regulated at 49 and 60 dpc and down-regulated at 77 dpc for Lantang.

To gain further insight into the biological functions of the DE genes identified, GO analyses were preformed (Table S8, Figure S4, S5, S6, S7). We chose significant GO categories with P-value <0.05. The muscle development-related biological processes are presented in Figure 3. Genes related to muscle development, including muscle cell development and differentiation or myofibril assembly (Table S8) were up-regulated at 49 dpc in LT, 63 dpc in LR and down-regulated at 77 dpc in both breeds. In addition, genes related to muscle system processes and muscle contraction (Table S8) were up-regulated during early fetal stages such as 49 and 63 dpc, and down-regulated at 77 dpc in both breeds. Moreover, the immune and nervous systems developed during early embryonic stages. During postnatal development, the main GO categories were related to metabolism and biosynthetic processes or energy.

#### Pathway analysis of temporal DE genes

All pathways of DE genes were summarized in Table S9, and the significant pathway categories (P <0.05) were listed Table 2, Figure S8 and S9. Based on the results of the GO analysis, we focus on the pathways of DE genes up-regulated at 35, 49 and 63 dpc and down-regulated at 77 dpc. The DE genes involved in *Wnt*, *Notch*, *TGF-beta*, insulin, calcium, chemokine and *GnRH* signaling pathways highly expressed at 35 dpc in LR, while only the *TGF-beta* and neurotrophin signaling pathways highly expressed at 35 dpc in LT. At 49 dpc, the DE genes involved in *PPAγ*, *MAPK* and calcium signaling pathways highly expressed in LR while *MAPK*, *GnRH*, neurotrophin, calcium, *ErbB* and *TGF-beta* signaling pathways highly expressed in LT. At 63 dpc, *MAPK*, *ErbB*, *TGF-beta*, *GnRH* and pentose phosphate signaling pathways highly expressed in LR while only the *PPAγ* signaling pathway highly expressed in LT. At 77 dpc, the DE genes involved in *TGF-beta*, neurotrophin and cell cycle signaling pathways were down-regulated in LR, while *Notch* signaling pathways were down-regulated in LT.

**Table 2:** Signaling pathways of DE genes.

Pathway	49/35 dpc		63/49 dpc		77/63 dpc		91/77 dpc		2 dpn/91 dpc		28/2 dpn		90/28 dpn		120/90 dpn		180/120 dpn	
	LR	LT	LR	LT	LR	LT	LR	LT	LR	LT	LR	LT	LR	LT	LR	LT	LR	LT
PPAR	↑			↑					↑	↑	↓	↑	↓	↓		↑	↑	↑
MAPK	↑	↑	↑		↑	↑	↓		↑	↑↓	↓	↑↓	↑	↑↓	↑↓	↑↓		↓
Calcium	↑↓	↑						↑	↑	↓	↓	↓	↓	↑	↓	↓		
Chemokine	↓									↓					↓	↓		
Wnt	↓								↓	↓	↓	↓		↑	↓	↓		
GnRH	↓	↑	↑	↓		↑		↓	↑	↓	↑	↓	↑	↑	↓	↓		
Insulin	↓			↓				↑	↑	↑	↓	↓	↓	↑↓	↓	↓	↑	↑
TGF-beta	↓	↑↓	↑	↓	↓	↑		↓	↓	↓	↓	↓			↑↓	↑↓		
Neurotrophin	↓	↑↓		↓	↓				↑	↓	↓	↓	↑	↑↓	↓	↓		
Notch	↓					↓	↓			↓		↓	↓					
Adipocytokine							↑		↑		↓		↓		↓		↑	
Pentose phosphate		↑		↑		↑			↑		↑	↑	↑	↑				↓
p53									↑	↑	↓	↓		↓				
VEGF									↑	↑	↑				↓	↓		
Jak-STAT									↓	↓			↓					
ErbB												↓	↑	↑			↓	
mTOR						↓									↓	↓		
Fatty acid metabolism		↑		↑						↑		↑		↑		↓		

#### STC and STC-GO analysis

In order to profile a gene expression time series and search for the most probable set of clusters generating this time series, the STC algorithm of gene expression dynamics was used, which took into account the dynamic nature of temporal gene expression profiles during clustering and identified the number of distinct clusters. Gene Ontology (GO) of significant STC cluster profiles was performed using the two-sided Fisher's exact test. We chose significant GO categories that had P<0.05. Approximately 480 types of temporal expression patterns with 133 significant cluster profiles were identified, of which 19 were related to muscle development and divided into several categories (Figure S11, Table S3, Table S4 and Table S5).

During the prenatal period, the DE genes involved in profile 2, 12 and 28 of LR, and profile 1 and 5 of LT highly expressed during early embryogenesis, and related to muscle development, cell growth or proliferation, and multicellular organismal development (Table S4 and Table S5). The DE genes involved in profiles 67, 73, 76 and 77 of LT, and profiles 67, 68, 76, 77 and 80 of LR were up-regulated at 49 or 63 dpc, and related to muscle development, differentiation and anatomical structure development, muscle contraction and muscle system processes, cell proliferation, cell differentiation and development, and immune system process (Table S4 and Table S5).

During the postnatal period, The DE genes involved in profiles 30, 32 and 39 of LR, and profile 2, 5 and 12 of LT predominantly highly expressed during early stages of development, and were related to the muscle development, muscle system or contraction muscle fiber development, muscle filament assembly or disassembly, muscle system process and muscle contraction, organ morphogenesis and tissue remodeling. In addition, the muscle development related profile 49 in postnatal LT was temporally up-regulated. (Table S4 and Table S5).

During the whole development process, The DE genes involved in profiles 71 and 64 of LR were related to muscle development, muscle contraction and muscle system process, genes involved in regulating cell proliferation, organelle organization. Profile 12 of LT was related to cell differentiation, system and organ development. Genes involved in profile 79 of LR and profile 79 of LT were predominantly related to muscle contraction and muscle system processes. (Table S4 and Table S5).

### Comparison of DE genes between the two breeds

About 3449 and 185 genes were differentially expressed in the two breeds during the prenatal and postnatal periods, respectively (log_2_ ratio>0.5, p<0.009, FDR<0.02). During prenatal period, 1179 were more abundantly expressed in LR, while 2268 were expressed at a higher level in LT. Postnatal, 48 were more abundantly expressed in LR, while 137 were expressed at a higher level in LT (Table S2). GO enrichment and pathway analysis of the DE genes were performed.

#### Gene Ontology comparison of DE genes between breeds

During prenatal period, GO analysis indicated that more development-related genes were highly expressed in LR while more enzyme, transport and location, metabolism and immune related genes were highly expressed in LT. During the postnatal period, genes involved in catabolic and metabolic processes, chromatin silencing, protein translocation, negative regulation of organ growth, negative regulation of nervous system development and negative regulation of adipose cell differentiation were differentially expressed (Table S8, Figure S10, P <0.05).

#### Pathway comparison of DE genes between breeds

Pathway analysis of prenatal DE genes demonstrated that those genes up-regulated in LR were related to metabolic pathways, muscle contraction and p53 signaling. In contrast, genes up-regulated in LT were related to muscle contraction, *MAPK*, neurotrophin, *Wnt*, calcium, *TGF-beta*, *mTOR*, *Jak-STAT*, *GnRH* and insulin signaling among others. Few significant different pathways could be identified between the two breeds postnatal (Table S9, Figure S10).

### Analysis of the DE genes related to muscle development

595 genes, probably related to muscle development, were identified by GO and pathway analysis (Table S6, S7, S8). More muscle-related genes were expressed at a high level between 35 and 63 dpc, and between 91 dpc and 90 dpn. Most muscle-related DE genes were identified during fetal development and the early stages after birth in both breeds.

#### The expression patterns of MRF and MEF2 families

**Figure 4:**
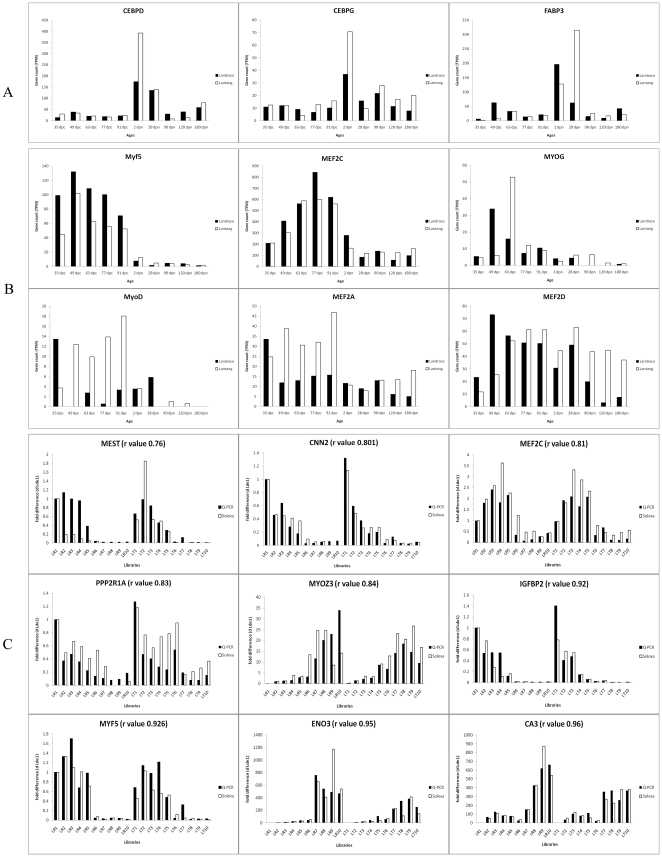
The expression patterns of myogenesis and adipogenesis genes (A, B) and Q-PCR validation of sequencing data (C). (A) The expression pattern of genes could relate to adipogenesis. The vertical axis indicates the normalized gene expression level in breed or stages; dpc indicates prenatal and dpn indicates postnatal. (B) The expression patterns of *MRF* and *MEF2* families. (C) Validation of sequencing data by Q-PCR. The vertical axis indicates the fold changes of transcript abundance in 20 samples compared to the LR 1. The r value indicates Spearman's Correlation between the two methods.

**Figure 5:**
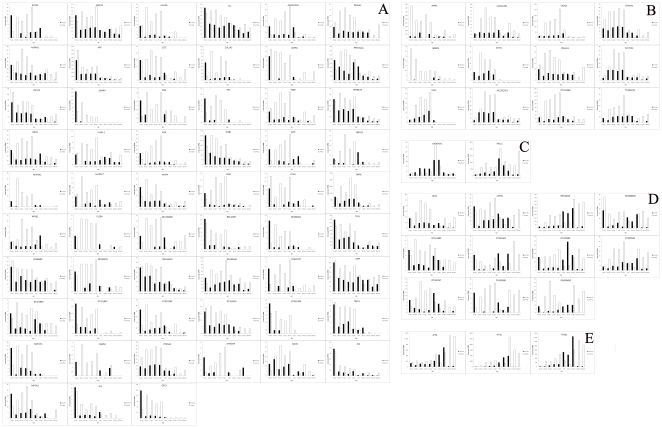
The muscle developmental genes which were higher expressed in LT than in LR. (A) These 53 genes were similar expressed with *MyoD*; (B) These 10 genes were only highly expressed in prenatal LT; (C) *GADD45G* and *FHL1* were peak expressed at 2 dpn and 28 dpn; (D) These 10 genes were similar expressed with *MyoD* in prenatal, but highly expressed at 2, 28 and 90 dpn of landrace while 90, 120 and 180 dpn of Lantang. (E) The DE genes could relate to muscle development in postnatal between the two breeds.

**Figure 6:**
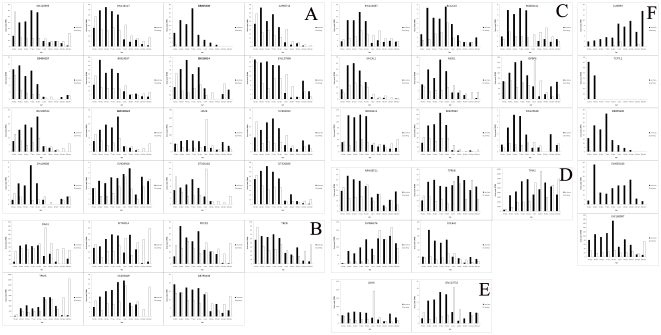
The muscle developmental genes which were higher expressed in LR than in LT. (A) These 13 genes were highly expressed at 35 dpc of the two breeds, and then maintained the high expression in prenatal LR but down regulated in prenatal LT; (B) These 7 genes were similar expressed with MEF2D; (C) These 9 genes were highly expressed in prenatal of the two breeds, but higher expressions were showed in LR than in LT; (D) These genes were differential expressed in prenatal between the two breeds, but showed no differences in postnatal; (E) LSM4 and DN123732 were highly expressed in prenatal LR and 2 dpn of LT; (F) These 5 genes were only highly expressed in LR.

The most important genes for muscle development are the myogenic regulatory factor (*MRF*) and myocyte enhancer factor 2 (*MEF2*) families, which are central to the regulation of myogenesis. However, their differential expression has not been reliably detected in previous expression profile research [3], [6], [19]. In present study, all *MRF* members including *Myf5*, *MyoD*, *MYOG* and *MYF6*, and three members of the *MEF2* family, *MEF2A*, *MEF2C* and *MEF2D*, were detected differential expression. Similar expression patterns were found in both breeds for *Myf5*, *MYOG* and *MEF2C*, and different patterns were identified for *MyoD*, *MYF6*, *MEF2A* and *MEF2D* (Figure 4). The expression of *MyoD* peaked at 35 dpc and then down-regulated in LR, but up-regulated at 49 dpc and maintained a high level of expression until 91 dpc in LT. Several genes had similar expression patterns with *MyoD* including myogenesis activators and myogenesis inhibitors (*ID1*, *ID2*, *CABIN1*, *MSTN*, *SMAD4*, *CTNNA1*, *NOTCH2*, *GPC3* and *HMOX1*) (Figure 5A). *Myf5* was up-regulated from 35 dpc, its expression peaked at 49 dpc and kept high level throughout fetal development in both breeds. Several myogenesis activators had similar expression patterns to *Myf5* (Figure S12). The expression of *MYOG* peaked at 49 dpc in LR and at 63 dpc in LT, but lowly expressed during other stages in both breeds. The expression pattern of *MEF2A* was similar to *MyoD*, while *MEF2C* was similar to *Myf5*. *MEF2D* was up-regulated at 49 dpc and maintained high expression until 90 dpn in LR, while it was up-regulated at 63 dpc and then maintained high expression throughout development in LT. Several myogenesis genes also showed similar expression pattern with *MEF2D* (Figure 6B).

#### The muscle developmental DE genes between different developmental stages

The expression patterns of the 595 muscle developmental genes during different stages were analyzed (Table 1 and Table S6). Most myogenesis genes were up regulated or highly expressed at 35, 49, 63 dpc and at 2, 28 dpn, including *MRF* and *MEF2* families, most myogenesis inhibitors (*ID1* and *ID2*, *CABIN1*, *MSTN*, *SMAD4*, *CTNNA1*, *NOTCH2*, *GPC3*, *HMOX1*, *IKBKB*, *ACVR1* and *ITGA*) and most activators (Table S6).

In postnatal, muscle phenotype variation was apparent during the periods from 91 dpc to 28 dpn (Figure 1). As expected, several muscle developmental genes were also showed differential expression during this period (Table S6). But, few muscle developmental genes could be identified during the postnatal period after 28 dpn except some genes related to muscle structure, contraction and metabolism (Table S6).

#### The muscle developmental DE genes between the two breeds

During prenatal period, 109 genes were related to muscle development among 2268 DE genes which more highly expressed in LT than in LR (Table S6, Figure 5). The expression patterns of 56 genes were similar with *MyoD* including 10 myogenesis inhibitors (*ID1* and *ID2*, *CABIN1*, *MSTN*, *SMAD4*, *CTNNA1*, *NOTCH2*, *GPC3* and *HMOX1*) (Figure 5A). 10 of the 109 genes only highly expressed in prenatal LT (Figure 5B). The expression level of *GADD45G* and *FHL1* peaked at 2 dpn and 28 dpn (Figure 5C). Another 11 of the 109 genes were similar expressed with *MyoD* in prenatal, but highly expressed at 2, 28 and 90 dpn of LR while 90, 120 and 180 dpn of LT (Figure 5D). In addition, 58 genes were related to muscle development among 1179 DE genes which more highly expressed in LR than in LT (Table S6, Figure 6). 13 of these genes highly expressed at 35 dpc of the two breeds, and then maintained the high expression in prenatal LR but down regulated in prenatal LT (Figure 6A). 7 genes shared similar expression pattern with *MEF2D* (Figure 6B). 9 genes highly expressed in prenatal both two breeds, but higher expressions were showed in LR than in LT (Figure 6C). *TPM2*, *TPRKB*, *COL6A1* and *DV896376* differentially expressed in prenatal, but showed no differences in postnatal between the two breeds (Figure 6D). *LSM4* and *DN123732* highly expressed in prenatal LR and 2 dpn of LT (Figure 6E). *TCF7L2*, *CUGBP2*, *EW168087*, *DB805600* and *EW055180* only highly expressed in LR (Figure 6F).

During the postnatal period, most of the DE genes between the two breeds were related to muscle structure and contraction (Figure 5E), but very few muscle developmental DE genes were indentified (Table S6). It is interesting that *ATF4*, *MYL1* and *TNNI2* showed high expression during 2 dpn, 28 dpn and 90 dpn of LR, but showed high expression during 90 dpn, 120 dpn and 180 dpn of LT (Figure 5E).

#### The expression patterns of genes related to adipogenesis

During the prenatal period, the expression levels of adipogenic genes including *FABP3*, *CEBPD* and *CEBPG* were very low and no differences were found between the two breeds. However, these genes were up-regulated at 2 or 28 dpn when *MyoD* expressed lowly. These adipogenic genes more highly expressed in LT than LR (Figure 4). However, other important adipogenic genes including *PPARA* and *PPARG* expressed at very low levels in this study (Table S6). In addition, several adipose related genes including *UCP3*, *HSPB1* and *ANGPTL4*, and muscle metabolism genes including *ACADL*, *ACADM*, *FOS*, *CASQ1*, *ABRA*, *AK1*, *ENO3* and *PLN* also highly expressed at 2 or 28 dpn of two breeds but more highly expressed in LT than in LR (Table S6).

#### Signal-flow analysis of DE genes

**Figure 7:**
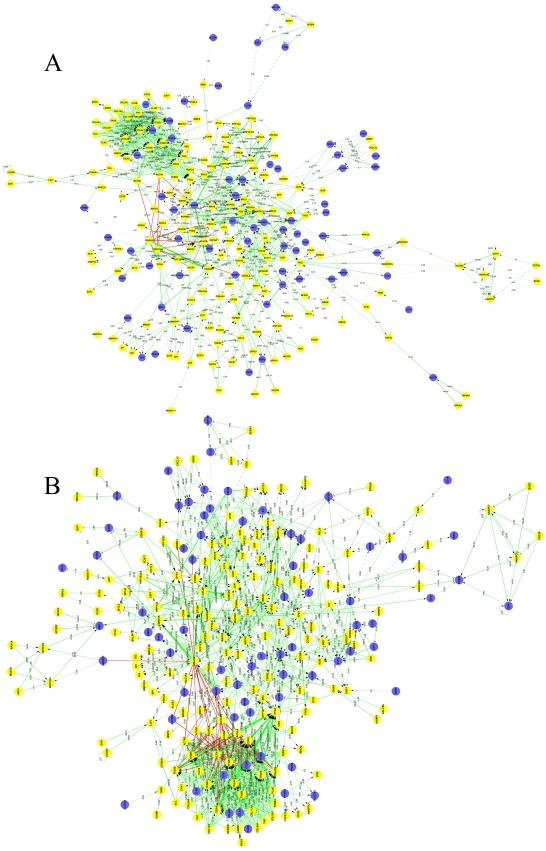
The interaction network between the differentially expressed muscle developmental genes. (A–B) Signal-flow analyses of myogenesis-related genes in LR (A) and LT (B). Yellow dots indicate DE genes and blue dots indicate non-DE genes. Straight lines indicate interaction relationships between genes; area indicates active and flat indicates inhibited. Solid line indicates a direct interaction and dashed line indirect interaction. Value and diameter of line indicates the size of interaction; the larger the values the stronger the regulation.

To investigate the key genes in muscle development and their interaction, regulatory network maps were constructed on the basis of muscle development signal pathways and the novel possible interactions obtained from these sequencing data. Approximately 237 and 258 genes were included in the signal flow maps for LR and LT, respectively. Among the signal flow maps, 763 and 909 potential direct interactions between differentially expressed genes were identified for LR and LT, respectively. Among these potential direct interactions, 604 of LR and 715 of LT were related to known muscle genes (Figure 7, Table S7). Moreover, *GSK3B* appeared to be more important in LR than in LT, while *SMAD4* appeared more important in LT than in LR. The potential interactions between the myogenesis genes were identified using signal-flow analysis.

### Confirmation of Solexa expression patterns using Real-time quantitative PCR

In order to validate the results of sequencing, nine muscle development-related genes were chosen for real-time quantitative PCR analysis (Figure 4); the results were presented as fold changes in expression normalized to the *AK239456.1* or *AK231315.1* gene and relative to the LR1 sample. Spearman's correlation coefficient (r) indicated that DGE and qPCR data had high consistency and the r values ranged from 0.76 (*MEST2*) to 0.96 (*CA3*) between the two methods.

## Discussion

The molecular mechanism underlying muscle development in pig is complex and remains unclear [3], [7], [25]. Here, we present the first genome-wide research of longissimus dorsi muscle from embryo to adult in two pig breeds using the Solexa/Illumina system, a novel tag-based high-throughput transcriptome deep sequencing method.

### Analysis of sequencing data

Approximately 4.2 million tags per library were identified (Table S1). Very high quality sequencing provided sufficient sequence data for the detection of low abundance and novel genes [22]. The distribution of tags demonstrated that most genes expressed at low levels (Table S1, Figure S1 and S2). Therefore, large amounts of information may be lost in similar studies using Microarray or LongSAGE. Tag mapping and gene annotation analysis demonstrated that many tags were not mapped to the reference tag database and several were mapped to antisense genes (Table S1). This indicated that the DGE system provides an unbiased analysis of the transcriptome. And the qPCR confirmation results indicated that the Solexa sequencing was reliable.

### New functions and regulations of *MRFs* and *MEF2* families during muscle development

The *MRFs* and *MEF2* families are key genes in the regulation of myogenic determination and differentiation [15], [16], [26]. Similar studies using Microarray have demonstrated that *MRFs* are unchanged during porcine myogenesis and that myogenesis in pig might depend on the balance of differentiation-activators and differentiation-inhibitors [6], [19]. However, in the present study, changes in *MRFs* and *MEF2* families during muscle development were identified owing to the high resolution of Solexa sequencing.

**Figure 8:**
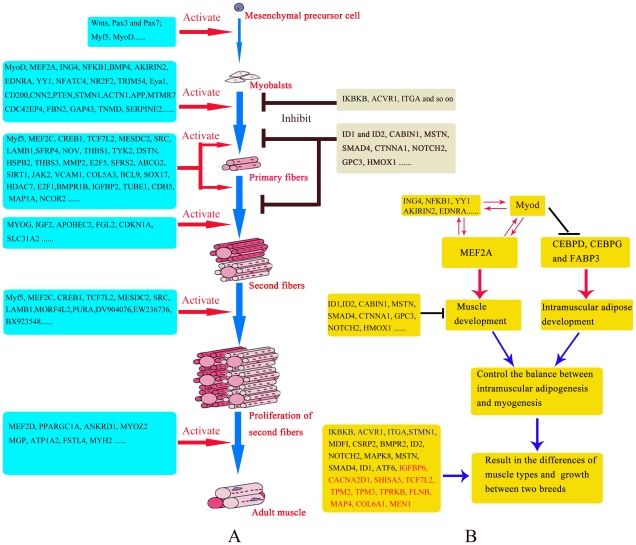
The molecular regulation of myogenesis and the model for *MyoD* controls intramuscular adipogenesis and myogenesis. Red dots indicate the promoting roles of these genes in myogenesis and blank striping indicate the repressing roles. (A) The probable roles of DE genes in regulating myogenesis (B) The novel model for *MyoD* controls the balance between intramuscular adipogenesis and myogenesis.

Compared with other reports, the present study reveals a new expression pattern for *MyoD* during muscle development. The expression of *MyoD* was very low except 35 dpc in LR, while high throughout the fetal stage and low after 2 dpn in LT (Figure 4). The high expression during early embryogenesis in both breeds indicated that *MyoD* is necessary for myogenic determination [15], [16], [26]. Some reports have demonstrated that forced expression of *MyoD* in vitro strongly induces myogenic differentiation while inhibiting adipocyte differentiation [27]. Our results show that adipogenic factors including *CEBPD* and *CEBPG* expressed at very low levels while *MyoD* highly expressed during the fetal period. Furthermore, *CEBPD* and *CEBPG* were up-regulated at 2 dpn while *MyoD* expressed at low level (Figure 4). This suggests a possible negative correlation between the *CEBP* family and *MyoD*. Furthermore, *CEBPD* and *CEBPG* might be repressed during the fetal period, and more repressed in LT because *CEBPD* and *CEBPG* higher expressed in LT than in LR. This might be the reason for the expression of *MyoD* was higher in LT than LR during fetal development. Considering the expression patterns of *MyoD*, *CEBPD* and *CEBPG*, *MyoD* might be critical in promoting myogenesis and inhibiting intramuscular adipose differentiation by repressing adipogenic factors including *CEBPD* and *CEBPG*. This hypothesis could explain not only the differential expression of adipocyte differentiation factors and *MyoD* in the two breeds but also the higher intramuscular fat content in LT than in LR (Figure 8). In addition, the genes involved in *Wnt* and *p53* signaling pathways might contribute to this regulation pattern of *MyoD* because these genes also higher expressed in prenatal LT than in LR (Table 2), and the roles of *Wnt* and *p53* signaling pathways in myogenesis by regulating *MyoD* have been demonstrated [28], [29].

Several genes had similar expression patterns with *MyoD* (Figure 5). The most important was *MEF2A*, which may have a similar function during myogenesis by interacting with *MyoD*, as direct interactions between *MRFs* and *MEF2* families have been observed [30]. Myogenesis inhibitors (such as *ID1* and *ID2*, *CABIN1*, *MSTN*, *SMAD4*, *CTNNA1*, *NOTCH2*, *GPC3* and *HMOX1*) had similar expression patterns to *MyoD* and *MEF2A* (Figure 5), which indicating that they might be important in preventing the excessive development of muscle caused by high expression of *MyoD* and *MEF2A* in LT. Among other genes with similar expression patterns to *MyoD* and *MEF2A* (Figure 5), *ING4* and *NFKB1* could bind *TP53* or *EP300/p300* in mammal cells which are co-activation factors of *MyoD* [31], [32], [33]. *AKIRIN2* performs a multifaceted role during vertebrate myogenesis and forms part of a module of co-expressed genes involved in muscle differentiation including *MyoD*, *MYOG* and *MEF2A* [34]. *EDNRA* is directly activated by downstream targets of the *WNT/beta-catenin* pathway in Wilms tumors [35], and WNT/beta-catenin pathway could regulates the function of MyoD and myogenin in myogenesis [36]. YY1 plays an repression role in regulating skeletal muscle differentiation by interfering with MyoD [37]. These genes could function during myogenesis by interacting with MyoD (Figure 5, Figure 8).

*Myf5* could regulate myoblasts proliferation [9], [38]. Both the primary and the secondary waves of differentiation have such a proliferation phase [9], [18], so *Myf5* was highly expressed during all embryonic stages in both breeds (Figure 4). The expression of *myf5* was higher in LR than LT, which might regulate the excessive muscle development in LR than in LT. Several genes had similar expression patterns to *myf5* (Figure S12). The most important is *MEF2C*, which presented with a similar expression pattern but was activated later (Figure 4). This indicated an interaction between *MEF2C* and *Myf5* in promoting myogenesis, as a direct interaction between *MRFs* and *MEF2* families in promoting myogenesis has been established [30]. Among other genes with similar expression to *Myf5* and *MEF2C* (Figure S12), many have been proven to have a role in myogenesis. *CREB* is required for *Wnt*-regulated processes in inducing the expression of myogenic determinant genes *Pax3*, *MyoD* and *Myf5* during mammalian embryogenesis [18], [38], [39]. *TCF7L2* and the *Wnt* signaling pathway are important in patterning the vertebrate limb myogenic differentiation [40], and *Myf5* is one of *Wnt* target genes [7]. *MESDC2* is also critical in modulating *Wnt* signaling [41]. *LAMB1* has been implicated in a wide variety of biological processes including mouse myogenesis [42], and its differential expression during muscle development has been observed in another study [19]. It is likely that these genes play roles during myogenesis by interacting with *myf5* (Figure 8).

The expression of *MYOG* peaked at 49 dpc in LR and 63 dpc in LT, while was low at other stages in both breeds (Figure 4, 8), indicating that *MYOG* contributes to the differentiation of secondary fibers and its earlier differentiation in LR than LT. *MEF2D* was up-regulated at 49 dpc and maintained high expression until 90 dpn in LR, while up-regulated at 63 dpc and then maintained high expression in LT (Figure 4, 8), indicating that *MEF2D* contributes to muscle growth, and also reflecting the slower growth of muscle in LT than in LR (Figure 6).

### The molecular regulation of myogenesis, muscle growth and maturation

Temporal GO analysis of the DE genes demonstrated that the genes involved in myogenesis GO terms were up-regulated at 49 dpc of LT and 63 dpc of LR while down-regulated at 77 dpc of the two breeds (Figure 3). These results indicated that the main process of myoblasts determination and proliferation might be carried out before 77 dpc, and the increased number of muscle fibers between 77 and 91 dpc might be due to the fusion of these myogenic cells. Moreover, more myogenesis genes higher expressed at 35 and 49 or 63 dpc which indicated the main myogenesis process were occurred during 35 dpc to 77 dpc (Table S6). In addition, histological examination demonstrated that the primary fibers were observed at 35 and 49 dpc in LT and LR respectively, while the secondary fibers formed before 63 dpc in both breeds (Figure 1). These results indicated that the genes highly expressed at 35 and 49 dpc are probably related to the differentiation of primary fibers while those highly expressed at 63 dpc are related to the differentiation of secondary fibers. Similar studies have demonstrated that 35 and 63 dpc are critical stages during myogenesis [6]. And in the pathway analysis of the DE genes, the genes involved in myogenesis pathways such as calcium [29], *Wnt* [29], *p53* [28] and Insulin signaling pathway [43] also highly expressed at 35, 49 or 63 dpc (Table 2).

Several DE genes were detected during these developmental stages, but some were not observed with SAGE or Microarray; for example, genes involved in the regulation of transcription, cell migration and adhesion, extracellular structure organization, cell cycle regulation, immune activities, nervous system, circulatory system, and organ and anatomical structure (Figure 3, Table S2 and Table S6). This study identifies many of these genes as being differentially expressed between obese and lean pig breeds for the first time.

During primary fiber differentiation, besides *Myf5/MEF2C* and *MyoD/MEF2A*, other myogenesis genes including *BMP4*, *NFATC4*, *NR2F2*, *TRIM54*, *Eya1*, *CD200*, *CNN2* and *PTEN* highly expressed at 35 and 49 dpc in both breeds (Table S2 and Table S6). *BMP4* could permits the muscle population expansion by preventing premature myogenic differentiation in muscle satellite cells [44]. *NFATC4* could control the muscle fiber type specification and modulate the phenotype and performance of skeletal muscle [45]. *NR2F2* (also known as *COUPTFII*) is required in early limb bud outgrowth and critical for appropriate development of the skeletal musculature of developing limbsis [46]. Expression of *TRIM54* (also known as *MURF*) is essential for skeletal myoblast differentiation and myotube fusion [47]. *Eya1* is required for hypaxial somitic myogenesis in the mouse embryo [48]. *CD200* (also known as *MOX1* or *MOX2*) is expressed in the paraxial mesoderm and is essential for normal vertebrate muscle formation and normal regulation of myogenic genes such as *Pax3* and *Myf5* [49]. Taken together, these data suggest that genes highly expressed at 35 and 49 dpc in both breeds might regulate myogenic cells proliferation and differentiation (Figure 8). In addition, myogenesis genes including *IKBKB*, *ACVR1* and *ITGA* highly expressed at 35 dpc in LR and 49 dpc in LT (Table S2 and Table S6). *ACVR1* belongs to the transforming growth factor (TGF)-beta super-family, which inhibits muscle differentiation [50]. *IKBKB* could regulate skeletal myogenesis via a signaling switch to inhibit differentiation and promote myotube homeostasis [51]. These genes highly expressed at 35 dpc in LR and 49 dpc in LT might regulate myogenic inhibition and promote myoblast proliferation, which contribute to proponed muscle differentiation and could explain the more muscle fiber numbers in LR than LT (Figure 8). In contrast, *STMN1*, which highly expressed at 35 dpc in LT and at 49 dpc in LR and controls the proliferation of C2 myoblasts and their differentiation [52], might contribute to the earlier differentiation and fewer muscle fiber numbers in LT than LR (Figure 8 and Table S6). However, many genes involved in muscle development highly expressed at 35 or 49 dpc in both breeds including *APP*, *CDC42EP4*, *FBN2*, *GAP43*, *TNMD*, *SERPINE2* and *MTMR7* (Table S6), but their roles in myogenesis are still unclear.

Besides *MYOG*, few myogenesis genes highly expressed at 63 dpc including *IGF2*, *APOBEC2*, *FGL2*, *CDKN1A* and *SLC31A2* (Table S2 and Table S6). It has been demonstrated that *APOBEC2*-deficient mice harbor a markedly increased ratio of slow to fast fibers, indicating that *APOBEC2* might contribute to secondary muscle fiber differentiation [53]. Similarly, *CDKN1A* (p21) also regulates slow and fast muscle differentiation [54]. *IGF2* expressed at high levels in lean pig breed, which promotes primary fiber differentiation [3]. However, in this study, high expression was observed at 49 dpc in LR and 63 dpc in LT (Table S2 and Table S6), indicating that *IGF2* might promote secondary as well as primary fiber differentiation. Taken together, these results suggest that those genes highly expressed at 63 dpc might contribute to the differentiation of secondary muscle fibers (Figure 8).

Some myogenesis genes highly expressed during the whole fetal stages (Table S2 and Table S6). These genes might be important throughout myogenesis (Figure 8). In addition, *MYH3* and *DLK1* highly expressed in the fetal stages of both breeds but higher in LT than in LR (Table S2 and Table S6). This might be related to the more oxidative fiber numbers in LT than LR [2], [55], [56]. Other genes related to muscle highly expressed during the fetal stages of both breeds including *IGFBP2*, *TUBE1*, *CDH5*, *MAP1A*, *BMPR1B* and *NCOR2* (Figure 8, Table S2 and Table S6).

In histological examination results, muscle phenotype variation was apparent during the periods from 91 dpc to 28 dpn (Figure 1). Several DE genes (few were related to myogenesis) were identified during the postnatal period, particularly from 91 dpc to 28 dpn (Table S2 and Table S6). Moreover, there is a fiber type transition and muscle maturation process from birth to the age of two months [14].Therefore, we speculated that the period from birth to 28 dpn could be critical for fiber transition and maturation. The probable role of *MEF2D* in this process has been discussed above (Figure 8). Several other myogenesis genes highly expressed during these periods (Table S2 and Table S6). *FHL1* has been identified as a regulator of skeletal muscle mass [57]. *PPARGC1A* was considered as a functional candidate gene for muscle fiber type composition and meat quality in pig [58]. *ANKRD1* and *MYOZ2* were found to be related to muscular dystrophy [59], [60]. These genes might contribute to the larger diameter of muscle fibers in LR than LT (Figure 1, Figure 8). *MGP*, *ATP1A2* and *FSTL4* highly expressed at 2 dpn in LR and 28 dpn in LT while *MYH2* highly expressed at 91 dpc in LT and 2 dpn in LR (Table S2 and Table S6, Figure 8).

### Molecular regulation underlying the differences in muscle growth and types between LR and LT

Primary fibers were observed at 35 dpc in LT but at 49 dpc in LR (Figure 1), which indicated that myoblasts were determined earlier in LT than in LR. Cagnazzo and his colleague attributed a prolonged proliferation of myoblasts in LR to delayed muscle fiber differentiation that results in increased primary muscle fibers [20]. In this study, more secondary muscle fibers were found in LR (Figure 1), which probably due to the more proliferation of myoblasts in LR than LT caused by the later muscle fiber differentiation. Meanwhile, temporal GO analysis of DE genes demonstrated that more myogenesis genes were up-regulated earlier in LT than in LR, but more myogenesis genes were up-regulated in 63 dpc of LR than in LT (Figure 3). These results could demonstrate the earlier but slower muscle development in LT than in LR, and lead to the less muscle mass in LT, which was probably the reason for the more fatness in LT. Myogenesis inhibitors including *IKBKB*, *ACVR1* and *ITGA*, which highly expressed at 35 dpc in LR and 49 dpc in LT, might also contribute to later muscle differentiation and the presence of more muscle fibers in LR than in LT (Figure 8, Table S2 and Table S6). In contrast, *STMN1* controls the proliferation of cells and their differentiation [52], highly expressed at 35 dpc in LT and 49 dpc in LR, which might contribute to the earlier differentiation and fewer muscle fiber numbers in LT (Figure 8, Table S2 and Table S6). In addition, the highly expressed myogenesis inhibitors (*ID1* and *ID2*, *CABIN1*, *MSTN*, *SMAD4*, *CTNNA1*, *NOTCH2*, *GPC3* and *HMOX1*) in LT might contribute to the slower muscle development (Figure 8, Table S6).

Histological examination showed that the cross-section areas of muscle fibers in LR were larger than in LT during postnatal growth (Figure 1). Cluster analysis and correlation analysis of 20 skeletal muscle libraries demonstrated that the expression pattern differences between the two breeds were larger during the prenatal than the postnatal periods, with the exception of 35 dpc (Figure 2). This indicated that the muscle phenotypic variation between the two breeds were probably determined from 49 to 91 dpc. The expression patterns of myogenesis genes were similar at 35 dpc but varied during 49 and 63 dpc between the two breeds (Table S6), indicating that the periods from 49 to 63 dpc (secondary muscle fiber differentiation) were critical for muscle phenotype variation. The differential expression of *MRF* and *MEF2* families between the two breeds at 49 and 63 dpc might play a role in determining the postnatal muscle phenotype variation. In addition, the highly expressed myogenesis inhibitors (above description) in LT and activators (*BCL2L15*, *IGFBP6*, *CACNA2D1*, *SHISA5*, *TCF7L2*, *TPM2*, *TPM3*, *TPRKB*, *FLNB*, *MAP4*, *COL6A1* and *MEN1*) in LR, (Table S2 and Table S6), might promote the larger muscle cross-section area and more muscle fiber numbers in LR than LT (Figure 8). Among the myogenesis activators highly expressed in LT, most had similar expression patterns to *MyoD* and *MEF2A* (Table S6 and Figure 5); these may confer to the more intramuscular fat content in LT.

Among other muscle genes differential expressed between the two breeds in prenatal, *DLK1* is an imprinted gene regulating skeletal muscle plasticity. Conditional gene knockout and over-expression analyses suggested *DLK1* could inhibit the muscle cell proliferation and enhance muscle differentiation by up regulating the expression of *MyoD* [61]. However, in the present study, *DLK1* were higher expressed in prenatal LT than in LR, and up regulated with the muscle development in prenatal (Figure 5B). This contradiction indicates a potential new role of *DLK1* in the regulating muscle development. *SPARC*, predominantly secreted by mesenchymal parietal endoderm, specifically promotes early myocardial cell differentiation in embryoid bodies by enhancing the expression of *bmp2* and *nkx2.5* in embryoid bodies and fetal cardiomyocytes [62]. However, in the present study, *SPARC* were only highly expressed in prenatal LT (Figure 5B).

In postnatal development, cluster and correlation analyses indicated that the gene expression patterns between breeds were similar in the early postnatal period but different after 90 dpn. Histological examination also demonstrated the muscle grew more slowly in LT than LR after 90 dpn (Figure 1–2). This might be caused by the more rapid fat deposition rate in LT than in LR. Most genes differentially expressed during these periods were related to muscle structure or metabolism. However, it is interesting that *ATF4*, *MYL1* and *TNNI2* showed a different expression pattern in the two breeds, which highly expressed in 2 dpn, 28 dpn and 90 dpn of LR but 90 dpn, 120 dpn and 180 dpn of LT (Figure 5E). *ATF4* could directly interact with helix-loop-helix domain of *bHLH* proteins (*MRFs* and *MEF2* family) to form heterodimers, and inhibit the actions of *bHLH* dimers on myogenic precursor cells [63]. And *MYL1* may play a role in myogenesis through the negative effect on the myoblast proliferation [64]. *TNNI2* is a muscle-specific myofibrillar proteins involved in calcium-sensitive regulation of contraction in skeletal muscle [65]. These results suggested their probably roles in inhibiting myogenesis and promoting muscle growth by negative interacting with myogenesis activators (such as *MRFs* and *MEF2* family). And these genes could contribute to the later and slower muscle growth in LT than in LR.

### The molecular regulation of intramuscular fat development

The *FABP3*, *C/EBP* and *PPAR* families are crucial for fat differentiation [66], [67]. We found that *CEBPD* and *CEBPG* highly expressed at 2 dpn of both breeds and *FABP3* highly expressed at 2 and 28 dpn, while *PPARA* and *PPARG* expressed at very low levels in skeletal muscle (Figure 4). This suggests that *FABP3*, *CEBPD* and *CEBPG* might be critical for intramuscular fat development while *PPARA* and *PPARG* contribute to subcutaneous fat development. The association between *FABP* genes and the intramuscular fat content has been extensively investigated by Gerbens [68], [69], [70]. In addition, *UCP3*, *HSPB1* and *ANGPTL4* highly expressed at 2 dpn in both breeds but higher expression was observed in LT (Table S6), indicating their association with intramuscular fat depots [71], [72]. The fatty acid metabolism genes were also up regulated from 2 dpn to 120 dpn of LT but not in LR (Table 2). These genes might contribute to intramuscular fat development by interacting with other muscle metabolism-related genes (*ACADL*, *ACADM*, *FOS*, *CASQ1*, *ABRA*, *AK1*, *ENO3* and *PLN*) (Table S6), and resulting in the more fatness of LT than LR.

### Identification of gene interaction networks during muscle development

Muscle development is a complicated biological process, switched and regulated by numerous interacting genes and series of signal transduction pathways. To investigate the key muscle development genes and their interactions, regulatory signal-flow network maps were constructed. Many potential interactions between genes related to muscle development were involved in the signal-flow networks (Figure 7, Table S7). These separate potential interactions were integrated into the regulatory network of muscle development on the basis of comparative transcriptome analysis. New potential interactions among DE genes in muscle development including *GSK3B*, *SMAD4*, *SMAD3*, *PPP1CB*, *ITGβ family*, *ACTN family*, *CDKN1A*, *ACADM*, *HRAS*, *NRAS*, *ACVR2A* and *ACVR2B* were identified (Figure 7, Table S7).

Among these potential interactions, it is very interesting that *GSK3β* had a more important role in the signal-flow network in LR than in LT. *GS3Kβ* is essential for specifying adipose of mesenchymal progenitor cells by regulating the balance between *β-catenin/Wnt* and *PPARγ* [73]. In the present study, the regulatory differences in *GSK3β* between the two breeds might contribute to the distinct early myogenesis process. Taken together, these data suggest that *GSK3β* might be more important in inhibiting primary fiber differentiation in LR and more important in inhibiting secondary fiber differentiation in LT, which might contribute to the earlier but slower differentiation of muscle fibers in LT than LR. Another myogenesis inhibitor, *SMAD4*, had a more important role in the signal-flow network of LT than LR. *SMAD4* could regulate the population expansion of myoblasts by preventing premature myogenic differentiation [44], and had a similar expression pattern with *MyoD* in the present study. The regulatory differences in *SMAD4* between the two breeds might also contribute to the distinct myogenesis process between the two breeds. Moreover, some new potential interactions between *SMAD4* and other myogenesis genes were identified in LT. Overall, a comprehensive transcriptional profile for myogenic differentiation has been constructed, which provides direction for studies concerning the molecular mechanism underlying muscle development.

In conclusion, we comprehensively studied the muscle developmental process from embryo to adult. We first exactly defined the various stages of muscle development, and identified a number of differentially expressed muscle development genes including new members during developmental stages and between breeds. The muscle differentiation processes during 49 dpc to 77 dpc were critical for formation of different muscle phenotypes. We also explained why muscle development began earlier and progressed more slowly in LT than in LR. And the earlier myogenesis and slower muscle development in LT than the LR might lead to the less muscle mass in LT, which might result in more fatness in LT. Most important, we further proposed a novel model in which *MyoD* and *MEF2A* controlled the balance between myogenesis and intramuscular adipogenesis by regulating the *CEBP* family; *Myf5* and *MEF2C* were essential during the whole myogenesis process while *MEF2D* affected muscle growth and maturation. These novel models were very different with previous study, and provided more exact regulation pattern of *MRFs* and *MEF2* family in myogenesis. Overall, this study contributed to research aimed at elucidating the mechanism underlying muscle development, and demonstrated its differences between the two breeds differing in muscle growth rate and fatness, which could provide valuable information for pig meat quality improvement.

## Materials and Methods

### Ethics Statement

All animal procedures were performed according to guidelines developed by the China Council on Animal Care and protocols were approved by the Animal Care and Use Committee of Guangdong Province, China. The approval ID or permit numbers are *SCXK (Guangdong) 2004-0011* and *SYXK (Guangdong) 2007-0081*.

### Preparation of experimental animals and tissues

Fifteen Lantang (LT) and 15 Landrace (LR) purebred sows with the same genetic background were artificially inseminated with semen from the same purebred boars. For each breed in prenatal ages, 2 sows per time point were slaughtered at dpc 35 (LT1, LR1), 49 (LT2, LR2), 63 (LT3, LR3), 77 (LT4, LR4) and 91 (LT5, LR5) after insemination, and embryos/fetuses were collected. The longissimus dorsi muscle tissues were dissected from all the embryos/fetuses. The longissimus dorsi muscle tissues from 3 male embryos/fetuses per time point were used as the experimental samples. For each breed in postnatal ages, 3 boars per time point were slaughtered at dpn 2 (LT6, LR6), 28 (LT7, LR7), 90 (LT8, LR8), 120 (LT9, LR9) and 180 (LT10, LR10) and longissimus dorsi muscle tissues from the same area were collected. All boars were castrated after two weeks. These samples were snap-frozen in liquid nitrogen and stored until further use or fixed in 10% neutralized buffered formalin for histological processing.

### Histology and histochemistry of muscle fibers

Muscle tissue samples were processed routinely for paraffin embedding and sections were cut and stained with hematoxylin and eosin, as described previously [74]. Micrographs were taken with an Axio Imager Z1 (ZEISS). Each area was photographed at a magnification of ×400.

### RNA extraction, library construction and Solexa sequencing

The RNA library was constructed and deep sequencing preformed by the Beijing Genomics Institute (BGI, commercial service). Total RNA was extracted from frozen muscle tissues using TRIzol reagent (Invitrogen) according to the manufacturer's protocols. RNA integrity and concentration were evaluated using an Agilent 2100 Bioanalyzer (Agilent Technologies, Palo Alto, CA, USA). For each time point, equal quantities of RNA isolated from three individual muscle tissues were pooled. Sequence tags were prepared using Illumina's Digital Gene Expression Tag Profiling Kit according to the manufacturer's protocols.

### Analysis of sequencing data

The raw data have been submitted to Gene Expression Omnibus (GEO) under series GSE25406. Clean tags were obtained by filtering raw data to remove adaptor tags, low quality tags and tags of copy number = 1. The clean tags were classified according their copy number in the library and the proportion of each category in relation to total clean tags was determined. Similar analyses were carried out for clean distinct tags. The saturation analysis of the sequencing library was completed by BGI.

### Tag mapping

All possible CATG+17-nt tag sequences (The “CATC site” is a digestion site of NlaIII enzyme. Due to most of the mRNA sequences (99%) have the NlaIII digestion site; therefore the NlaIII digestion site was used to produce the sequencing tags of Solexa which length is 21 bp (CATG+17 tags)) were created from the sus scrofa RefSeq and UniGene (NCBI36.1, 20090827) databases and used as reference sequences to align and identify the sequencing tags. All clean tags were aligned to the reference database, and then unambiguous tags were annotated. One mismatch in each alignment was allowed to tolerate polymorphisms across samples. Mismatch could be caused by a sequencing error, but the frequency is very low (1 or 2 per million).

### Cluster analysis and correlation analysis of 20 skeletal muscle libraries

Cluster 3.0 and TreeView software were used to analysis the systematic cluster of 20 libraries. And correlation analyses of 20 libraries were done by Pearson's correlation coefficient (SPSS software). The classes were defined by the similarity of these samples based on their genes expression level, which calculated by software such as Cluster 3.0 and SPSS. The “myogenesis genes” were defined by bioinformatics analysis. On the one hand, we extracted all muscle developmental genes from the entire muscle development related article (151959). On the other hand, the “myogenesis genes” were also selected based on the GO terms results of the sequencing data.

### Differential expression

To compare the differential expression of genes across samples, the number of raw clean tags in each sample was normalized to Tags per Million (TPM) to obtain normalized gene expression levels. Differential expression of genes or tags across samples was detected according to previously described methods [75]. Genes were deemed significantly differentially expressed with a P-value<0.005, a false discovery rate (FDR) <0.01 and an estimated absolute log_2_-fold change >0.5 in sequence counts across libraries.

### Real-time quantitative PCR

To validate the sequencing results, nine genes with differential expression were selected and analyzed using real-time PCR and the Lightcycler480 (Roche) with SYBR-Green detection (SYBR PrimeScript RT-PCR Kit, TaKaRa Biotechnology Co., Ltd.), according to the manufacturer's instructions. The samples used for real-time PCR assays were the same as those for DGE experiments. Real-time PCR was carried out on each cDNA and analyzed in triplicate, after which the average threshold cycle was calculated. The relative expression levels were calculated by the 2^−ΔΔCt^ method. Internal control genes such as GAPDH were also regulated during development. Therefore, we selected two genes that were relatively stable in two pig breeds as the reference genes (AK239456.1 and AK231315.1, coefficient of variation is 0.21). It is necessary to note that the real-time PCR experiments were only used to verify the sequencing data and no new data were produced.

### STC analysis

STC (Series Test of Cluster) is implemented entirely in java [76], [77]. The clustering algorithm first selects a set of distinct and representative temporal expression profiles. These model profiles are selected independently of the data. The clustering algorithm then assigns each gene passing the filtering criteria to the model profile that most closely matches the gene's expression profile as determined by the correlation coefficient. Since the model profiles were selected independently of the data, the algorithm can then determine which profiles have statistically significantly more genes assigned using a permutation test. This test determines an assignment of genes to model profiles using a large number of permutations of the time points. It uses standard hypothesis testing to determine which model profiles have significantly more genes assigned under the true ordering of time points compared to the average number assigned to the model profile in the permutation runs. Significant model profiles can either be analyzed independently or grouped together on the basis of similarities to form clusters of significant profiles. The numbers in every profile represents their expression patterns. The positive numbers represent up regulation and negative numbers represent up regulation. The size of the numbers represents the degree of change.

### GO analysis

GO, the key functional classification of NCBI, was applied to analyze the main functions of the differentially expressed genes. Fisher's exact test and a χ^2^ test were used to classify the GO category, and the false discovery rate (FDR) was calculated to correct the P-value; the smaller the FDR, the smaller the error in judging the p-value. We computed P-values for the GOs of all the differential genes. Enrichment provides a measure of the significance of the function: as the enrichment increases, the corresponding function is more specific, which helps to find those GOs with more concrete functional descriptions in the experiment [78].

### Pathway analysis

Pathway analysis was used to identify the significant pathways of the differential genes according to KEGG, Biocarta and Reatome. Fisher's exact test and a χ^2^ test were used to select each significant pathway, and the threshold of significance was defined by the P-value and FDR. The enrichment Re was calculated using the equation above [79].

### Signal-flow

External stimuli affect cellular behavior, as reflected in the protein interaction and gene expression kinetics. We inferred a dynamic gene regulatory network, which was calculated according to fold expressions and gene interaction in pathways. The relationships between the gene expression data were inferred using a continuous time recurrent neural network (CTRNN) as an abstract dynamic model for the gene regulatory network mediating the cellular decision to migrate upon an external stimulus. The model describes the mutual influences of genes and their stimulus response as dynamic elements, regardless of how such an interaction or stimulation is realized in concrete biological terms.

## Supporting Information

Figure S1 **Distribution of total clean tags and distinct clean tags.** The distribution of these tags indicated the heterogeneity and redundancy of mRNA. (A) Total clean tag distribution of 20 samples. (B) Distinct clean tag distribution of 20 samples.
(TIF)

Figure S2 **Distribution of tags and gene expression.** (A) Distribution of tags. Most tags were expressed at very low levels. (B) Saturation of DGE libraries. Saturation analysis of the capacity of libraries demonstrated that newly emerging distinct tags became gradually fewer as the total sequence tags increased in number when that number was large enough. (C) Effect of library size on the number of genes identified. The rate of increase of all genes identified and genes identified by unambiguous tags declined drastically as the size of the library increased. When the library size reached one million, library capacity approached saturation. (D) Distribution of gene expression. Most genes were expressed at very low levels. (E–F) The positions of tags. Ideally the tag is the 3′-most one, but for alternative splicing or incomplete enzyme digestion, the tag may be the 2nd or 3rd from the 3′-most one.
(TIF)

Figure S3 **Mapping of total clean tags and distinct clean tags.** (A) Total clean tag mapping of 20 samples. (B) Distinct clean tag mapping of 20 samples.
(TIF)

Figure S4 **GO pathways of DE genes up regulated in Landrace.** The DE genes were functionally classified according to GO biological processes. A P-value of <0.05 in the two-side Fisher's exact test was selected as the significance criterion. These DE genes were sorted by the enrichment of GO categories. The vertical axis is the GO category and the horizontal axis is the enrichment of GO.
(TIF)

Figure S5 **GO pathways of DE genes down regulated in Landrace.**
(TIF)

Figure S6 **GO pathways of DE genes up regulated in Lantang.**
(TIF)

Figure S7 **GO pathways of DE genes down regulated in Lantang.**
(TIF)

Figure S8 **Signaling pathways of DE genes in Landrace.** Pathway analysis was predominantly based on the KEGG database. A P-value of <0.05 and an FDR of <0.05 in the two-side Fisher's exact test were selected as the significance criteria. The vertical axis is the pathway category and the horizontal axis is the log_10_ (p Value) of these significant pathways.
(TIF)

Figure S9 **Signaling pathways of DE genes in Lantang.**
(TIF)

Figure S10 **GO and Signaling pathways of DE genes between the two breeds.**
(TIF)

Figure S11 **STC (Series Test of Cluster) analysis of DE genes.**
(TIF)

Figure S12 **Genes similarly expressed to Myf5 and MyoD.** (A) Genes with similar expression to Myf5. (B) Genes with similar expression to MyoD.
(TIF)

Table S1 **Major characteristics of 20 DGE libraries.**
(XLS)

Table S2 **Summary of differentially expressed (DE) genes identified.**
(XLS)

Table S3 **STC (Series Test of Cluster) analysis of DE genes.**
(XLS)

Table S4 **The muscle developmental STC-GO of Landrace.**
(XLS)

Table S5 **The muscle developmental STC-GO of Lantang.**
(XLS)

Table S6 **Distribution analysis of muscle developmental genes.**
(XLS)

Table S7 **Signal-flow analysis of muscle developmental genes.**
(XLS)

Table S8 **GO pathways of DE genes.**
(XLS)

Table S9 **Signaling pathways of DE genes.**
(XLS)
