# Supplementary figures and images for: Comparative Analyses by Sequencing of Transcriptomes during Skeletal Muscle Development between Pig Breeds Differing in Muscle Growth Rate and Fatness
Source: PLoS One. 2011 May 26;6(5):e19774. doi: 10.1371/journal.pone.0019774 (PMC3102668; doi:10.1371/journal.pone.0019774)

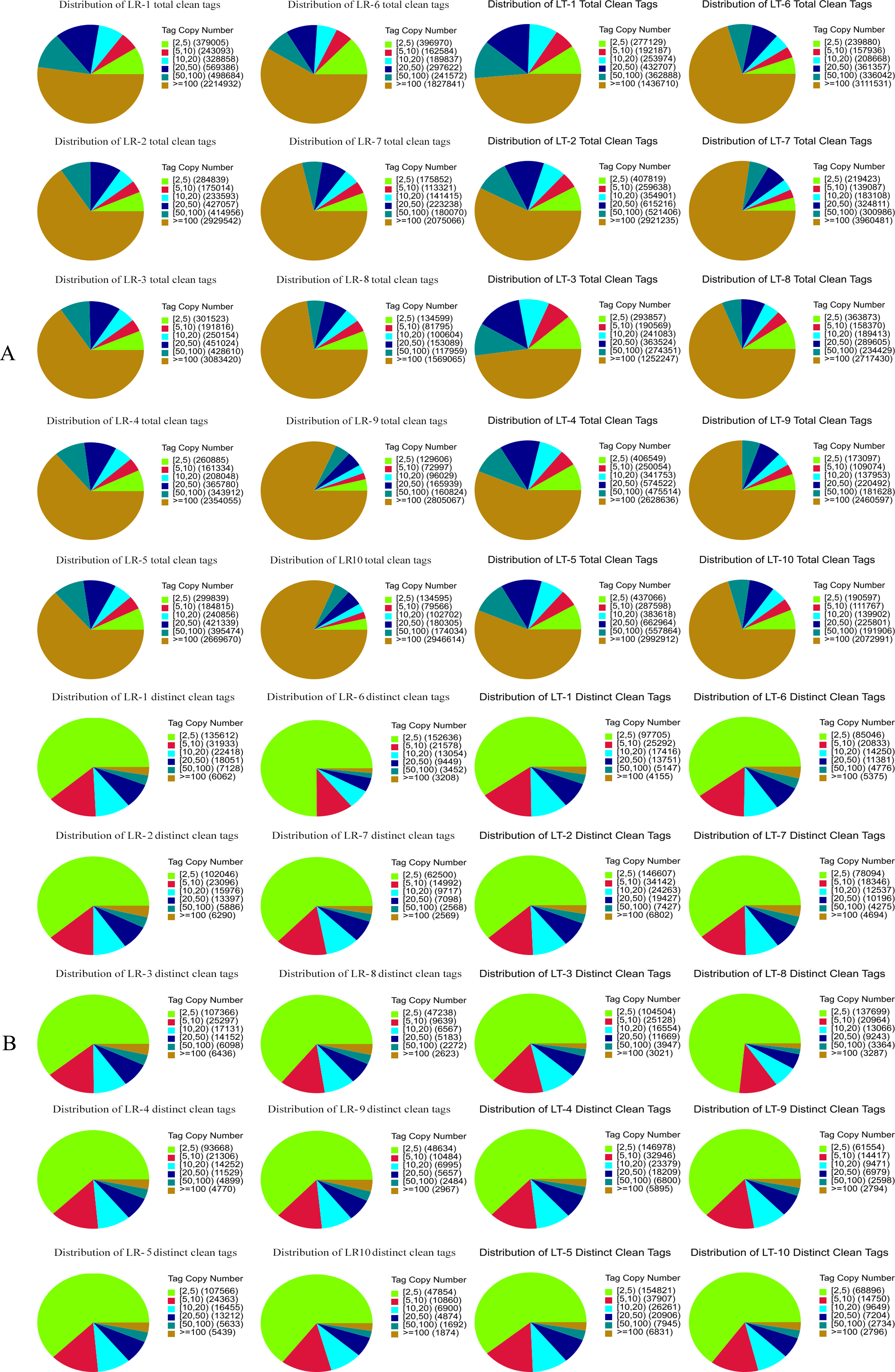

Supplement: Figure S1 — Distribution of total clean tags and distinct clean tags. The distribution of these tags indicated the heterogeneity and redundancy of mRNA. (A) Total clean tag distribution of 20 samples. (B) Distinct clean tag distribution of 20 samples. (TIF) [file pone.0019774.s001.tif]

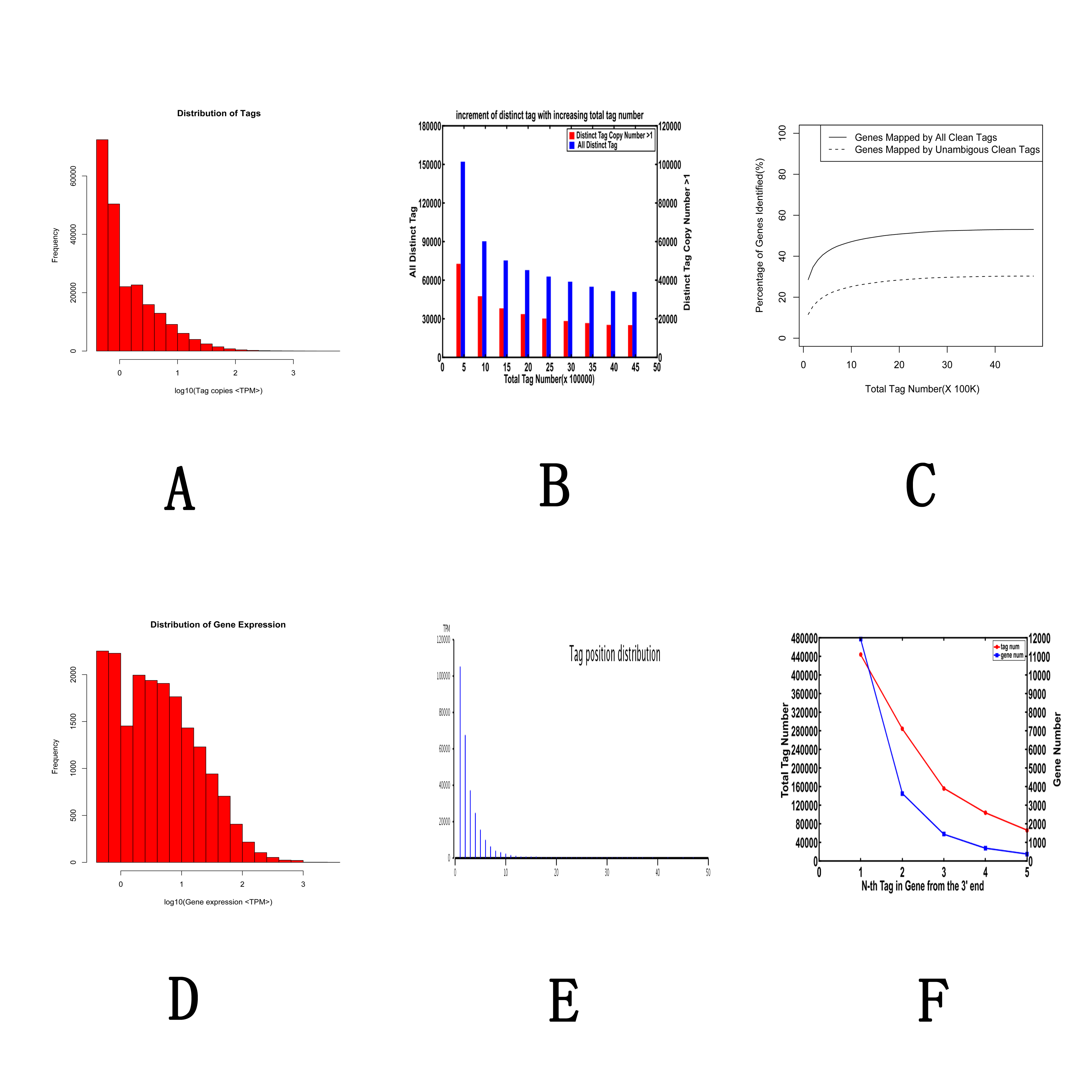

Supplement: Figure S2 — Distribution of tags and gene expression. (A) Distribution of tags. Most tags were expressed at very low levels. (B) Saturation of DGE libraries. Saturation analysis of the capacity of libraries demonstrated that newly emerging distinct tags became gradually fewer as the total sequence tags increased in number when that number was large enough. (C) Effect of library size on the number of genes identified. The rate of increase of all genes identified and genes identified by unambiguous tags declined drastically as the size of the library increased. When the library size reached one million, library capacity approached saturation. (D) Distribution of gene expression. Most genes were expressed at very low levels. (E–F) The positions of tags. Ideally the tag is the 3′-most one, but for alternative splicing or incomplete enzyme digestion, the tag may be the 2nd or 3rd from the 3′-most one. (TIF) [file pone.0019774.s002.tif]

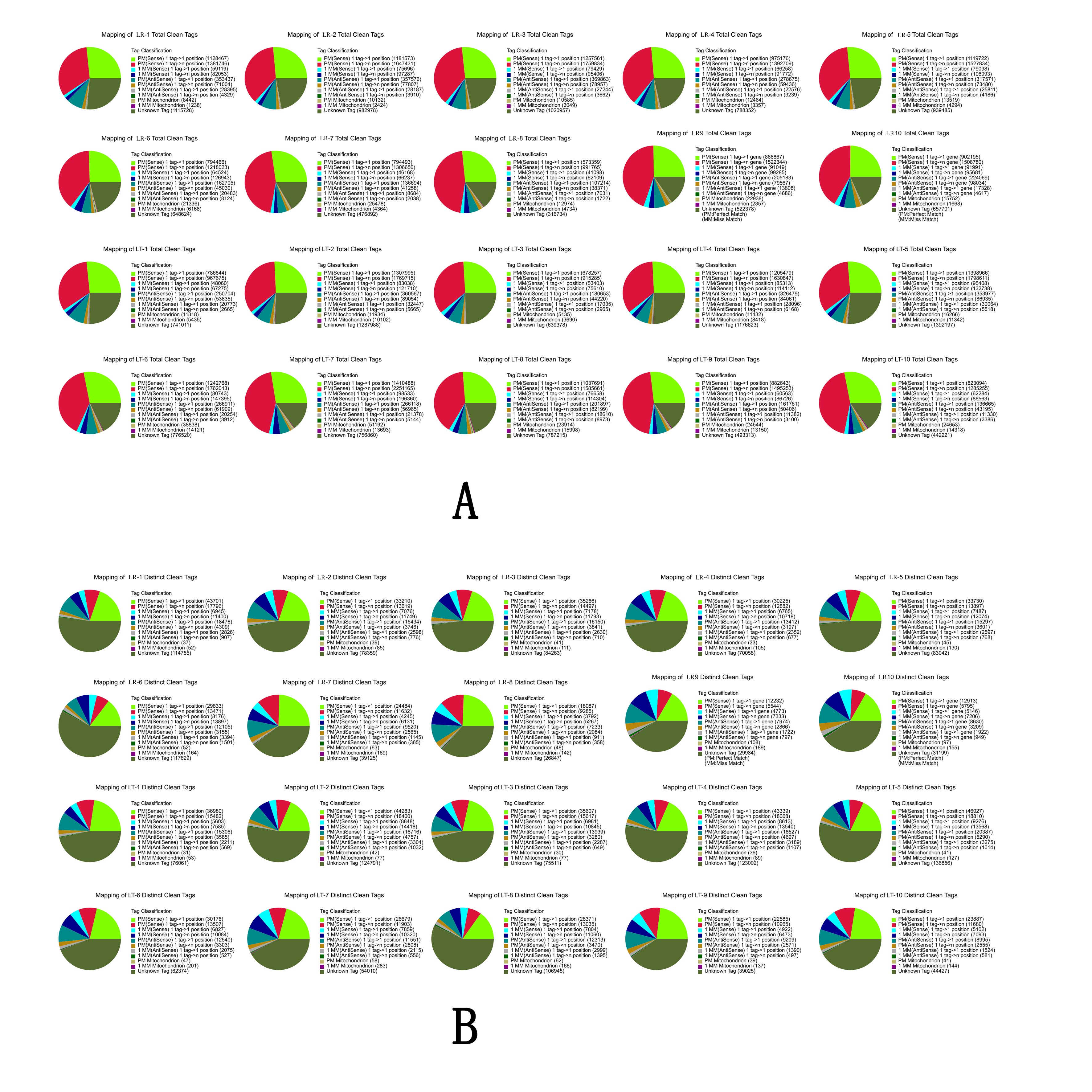

Supplement: Figure S3 — Mapping of total clean tags and distinct clean tags. (A) Total clean tag mapping of 20 samples. (B) Distinct clean tag mapping of 20 samples. (TIF) [file pone.0019774.s003.tif]

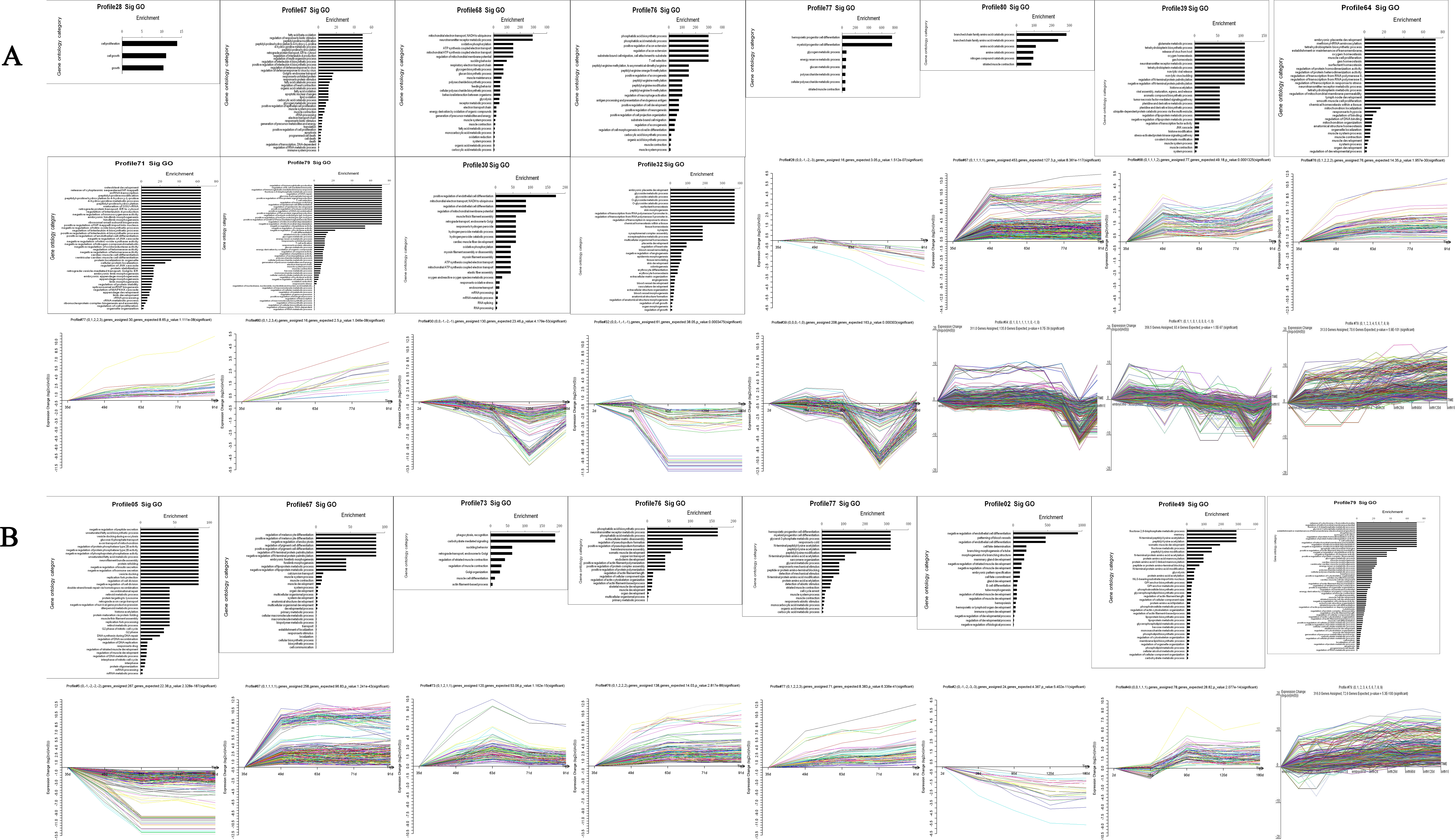

Supplement: Figure S11 — STC (Series Test of Cluster) analysis of DE genes. (TIF) [file pone.0019774.s011.tif]

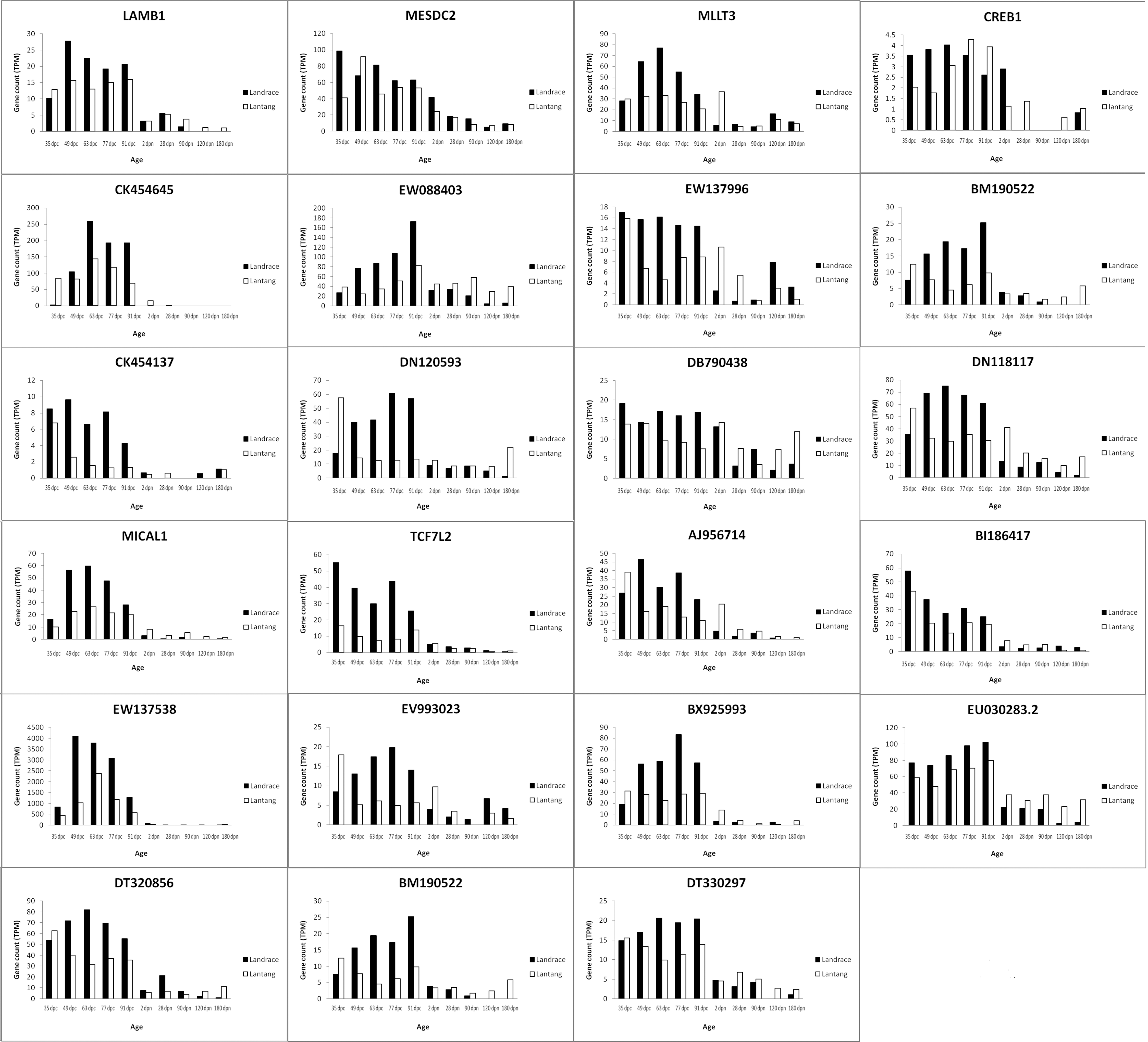

Supplement: Figure S12 — Genes similarly expressed to Myf5 and MyoD. (A) Genes with similar expression to Myf5. (B) Genes with similar expression to MyoD. (TIF) [file pone.0019774.s012.tif]
